# Evaluation of Quality of Life in Patients with and without Heart Failure in Primary Care

**DOI:** 10.5935/abc.20170123

**Published:** 2017-09

**Authors:** Antonio José Lagoeiro Jorge, Maria Luiza Garcia Rosa, Dayse Mary da Silva Correia, Wolney de Andrade Martins, Diana Maria Martinez Ceron, Leonardo Chaves Ferreira Coelho, William Shinji Nobre Soussume, Hye Chung Kang, Samuel Datum Moscavitch, Evandro Tinoco Mesquita

**Affiliations:** Curso de Pós-Graduação em Ciências Cardiovasculares - Universidade Federal Fluminense (UFF), Niterói, RJ - Brazil

**Keywords:** Heart Failure, Quality of Life, Primary Health Care

## Abstract

**Background:**

Heart failure (HF) is a major public health issue with implications on health-related quality of life (HRQL).

**Objective:**

To compare HRQL, estimated by the Short-Form Health Survey (SF-36), in patients with and without HF in the community.

**Methods:**

Cross-sectional study including 633 consecutive individuals aged 45 years or older, registered in primary care. The subjects were selected from a random sample representative of the population studied. They were divided into two groups: group I, HF patients (n = 59); and group II, patients without HF (n = 574). The HF group was divided into HF with preserved ejection fraction (HFpEF - n = 35) and HF with reduced ejection fraction (HFrEF - n = 24).

**Results:**

Patients without HF had a mean SF-36 score significantly greater than those with HF (499.8 ± 139.1 vs 445.4 ± 123.8; p = 0.008). Functional capacity - ability and difficulty to perform common activities of everyday life - was significantly worse (p < 0.0001) in patients with HF independently of sex and age. There was no difference between HFpEF and HFrEF.

**Conclusion:**

Patients with HF had low quality of life regardless of the syndrome presentation (HFpEF or HFrEF phenotype). Quality of life evaluation in primary care could help identify patients who would benefit from a proactive care program with more emphasis on multidisciplinary and social support.

## Introduction

Heart failure (HF) is a major public health issue with implications in health-related quality of life (HRQL).^1^ Patients with HF present limitations on their usual activities, suffering impairment on social interaction, with a progressive loss of physical autonomy. Signs and symptoms of HF have a strong impact on HRQL regardless of the phenotype, affecting patients with either preserved ejection fraction (HFpEF) or reduced ejection fraction (HFrEF). Although HFrEF and HFpEF differ regarding mortality and hospitalization rates,^2-4^ manifested signs and symptoms appear to have a similar impact on the well-being of those patients.^5^

To improve the HRQL of patients with HF is one of the major aims of the treatment. Additionally, many patients with HF usually attribute more importance to HRQL than to improvement in their survival.^6^

In the community setting, patients with HF are about a decade older, have multiple comorbidities and polypharmacy prescriptions, and are taking more medications than patients usually recruited for clinical trials.^7-9^ These patients may benefit from measures that may improve their HRQL.

The objective of the present study was to compare the HRQL, estimated by the Short-Form Health Survey (SF-36), in patients with and without HF, and between the two phenotypes, HFrEF and HFpEF, in the community.

## Methods

The Digitalis Study was a cross-sectional study including 633 volunteers, whose methodology is published elsewhere.^10^ Briefly, individuals aged 45 to 99 years, registered in the Family Doctor Program (PMF) of the city of Niterói, Rio de Janeiro State, Brazil, were randomly selected to attend community visits for examination. Data were collected from July 2011 to December 2012. Initially, the healthcare units of the PMF were randomly selected, proportionally to the number of individuals enrolled. Then, in each unit, individuals aged 45 to 99 years were also randomly selected.

For the present study, individuals were divided into two groups: group I, formed by HF^11^ patients (HF group - n = 59); and group II, formed by individuals without HF (n = 574). The HF group was divided into HFpEF (n = 35) and HFrEF (n = 24).

The Portuguese version of the SF-36 Questionnaire was used to classify HRQL.^12^

### Statistical analysis

Statistical analysis was performed with the SPSS software, version 21.0 (Chicago, Illinois, USA). Categorical variables were expressed as absolute numbers and/or percentages. Quality of life and its domains presented non-Gaussian distribution, thus, the differences between categories were presented as median and interquartile range, and the differences were tested with the non-parametric Mann-Whitney test. All comparisons were assessed with bilateral tests. A 5% statistical significance level was considered.

### Ethical considerations

This study was conducted in accordance with the principles of the Declaration of Helsinki, revised in 2000. The study protocol was approved by the Ethics Committee of the Institution under number 0077.0.258.000-10.

## Results

We evaluated 633 subjects (59.6 ± 10.4 years; 62% female; 63% black or brown skin-color). The HF patients were older, had lower educational levels, consumed less alcohol, and showed a higher prevalence of former smokers. The average overall score, bodily pain and general health perception differed between patients without HF as compared to patients with HF. Two dimensions, physical and emotional aspects, showed no variation (Table 1).

**Table 1:** Demographic characteristics and mean scores of the SF36 dimensions of individuals with and without heart failure

Variables	No HF (n = 574)	HF (n = 59)	p-value
**Age (years)**	58.4 ± 9.4	71.1 ± 12.4	< 0.001
**Female (%)**	61.8	61	0.901
**Never studied (%)**	5.4	17.2	0.020
**Family income (mean in US $)**	484.63 ± 461.71	406.85 ± 463.80	0.234
Black or brown skin-color****(%)	63.2	63.8	0.929
**Alcohol consumption(%)**	9.9	3.4	0.100
**Tobacco use**			0.012
Never smoker (%)	48.6	50.8	-
Former smoker (%)	31.2	42.4	-
Smoker (%)	20.2	6.8	-
**Private health insurance (%)**	15.3	15.3	0.949
**SF-36 dimensions**			
Physical functioning	85 (60-90)	55(25-80)	< 0.0001
Physical health	100(100-100)	100(100-100)	--
Emotional health	100(100-100)	100(100-100)	--
Vitality	70(50-85)	65(40-80)	0.01
Mental Health	80(60-92)	78(57-96)	0.265
Social functioning	100(62-100)	87(53-100)	0.296
Bodily pain	70(45-100)	80(49-100)	0.865
General health perception	70(50-85)	67(45-80)	0.091
Overall SF-36	535(403-615)	447(356-537)	0.001

HF: heart failure; P value (associated with two-tailed t test for independent samples); Chi-square test with continuity correction; median (interquartile range) with Mann-Whitney test for non-parametric variables.

Physical functioning was lower in patients with HF regardless of sex or age. Women, regardless of the presence of HF, scored lower for most of the dimensions than men did. The functional capacity - ability and difficulty to perform common everyday life activities -, general health perception and overall score were significantly worse in patients with HF independently of sex and age (Table 2).

**Table 2:** Mean SF-36 scores by sex and age in patients with and without heart failure

	Male (n = 242)			Female (n = 391)			Age (45 to 59 years) (n = 357)			Age (60 to 99 years) (n = 276)		
SF36 dimensions	No HF(n = 219)	HF(n = 23)	p-value	No HF(n = 355)	HF(n = 36)	p-value	No HF(n = 344)	HF(n = 13)	p-value	No HF(n = 230)	HF(n = 46)	p-value
Physical functioning	95(75-100)	55(23.7-81.2)	< 0.0001	80(55-95)	55(30-80)	< 0.0001	90(65-95)	60(22.5-77.5)	0.001	80(55-95)	55(25-80)	< 0.0001
Physical health	100(100-100)	100(100-100)	--	100(100-100)	100(100-100)	--	100(100-100)	100(100-100)	--	100(100-100)	100(100-100)	--
Emotional health	100(100-100)	100(100-100)	--	100(100-100)	100(100-100)	--	100(100-100)	100(100-100)	--	100(100-100)	100(100-100)	--
Vitality	80(60-90)	67.5(38.7-85)	0.05	60(45-85)	60(40-80)	0.571	70(45-85)	50(32.5-67.5)	0.037	75(50-90)	70(45-80)	0.215
Mental Health	84(72-96)	80(68-96)	0.455	76(52-88)	68(52-96)	0.987	76(52-96)	70(51-80)	0.086	80(60-92)	80(57-96)	0.891
Social functioning	100(75-100)	93.7(59.4-100)	0.228	100(62-100)	88(50-100)	0.419	100(75-100)	87.5(68.7-100)	0.582	100(62.5-100)	88(50-100)	0.260
Bodily pain	80(57-100)	80(61-100)	0.685	70(45-90)	80(42.5-100)	0.160	70(45-90)	57.5(27.5-80)	0.180	80(47.5-100)	80(57.5-100)	0.086
General health perception	70(55-85)	67(45-75)	0.058	70(50-85)	68(45-80)	0.568	70(55-80)	50(25-70)	0.027	70(55-85)	70(46.2-80)	0.265
Overall SF-36	585(488.2-632)	500.2(401.6-564.4)	0.003	497.7(367.9-591.4)	425(320.6-515.2)	0.056	537.5(412-615)	388(315-487.5)	0.005	529(386-615)	475(388.5-555)	0.058

HF: heart failure; median (interquartile range) with Mann-Whitney test for non-parametric variables.

Women had lower HRQL (vitality and general health perception) even in the absence of HF. Individuals younger than 60 years had a worse HRQL in the presence of HF, which was not observed in patients aged 60 years and older (Table 2).

Although the differences were not statistically significant (except for the vitality dimension), patients with HFpEF had lower mean values as compared to those with HFrEF (Table 3).

**Table 3:** SF-36 overall and dimension scores of individuals with heart failure with reduced or preserved ejection fraction (HFrEF and HFpEF, respectively)

SF36 dimensions	HFpEF (n = 35)	HFrEF (n = 24)	p-value
Physical functioning	55(25-77.5)	55(26.2-85)	0.582
Physical health	100(100-100)	100(100-100)	--
Emotional health	100(100-100)	100(100-100)	--
Vitality	55(36.2-70)	75(52.5-80)	0.024
Mental Health	68(44-96)	80(68-96)	0.143
Social functioning	93.7(50-100)	87.5(65.6-100)	0.951
Bodily pain	70(43.7-100)	100(58.7-100)	0.097
General health perception	62.5(45-80)	70(46.2-78.7)	0.420
Overall SF-36	441(314-520)	452(406-578)	0.126

Median (interquartile range) with Mann-Whitney test for non-parametric variables.

## Discussion

Patients with HF had a lower mean overall SF-36 score than patients without HF (53.1 ± 29.6 vs. 76.2 ± 24.9; p < 0.001). The HRQL worsening observed in this study was similar to data obtained in the literature.^13-15^

Age, vitality, pain and the overall SF-36 score were the four characteristics associated with worse HRQL in patients with HFrEF. On the other hand, only age was related to HRQL worsening in patients with HFpEF.

The CHARM study^16^ has evaluated the HRQL in HF patients and has concluded that those with HFpEF had a similar HRQL when compared to patients with low left ventricular ejection fraction (LVEF). That study showed that the extent of HRQL worsening was independent of LVEF. Our data did not show a difference between the overall SF-36 scores in patients with HFpEF and HFrEF (418.9 ± 122.5 vs. 476.6 ± 120.5; p = 0.101).

In general, older HF patients reported better quality of life than younger ones, regardless of the LVEF value. Studies have shown a better HRQL among older patients than among younger patients with HFrEF, although older patients had a worse functional status and performed worse in the six-minute walk test.^17^ Our data show that patients aged 45 to 59 years with HF have a more pronounced worsening of HRQL than those without HF (394.0 ± 106.4 vs. 501.3 ± 139.8; p = 0.012) when compared to patients aged ≥ 60 years (459.9 ± 125.7 vs. 497.7 ± 138.3; p = 0.113).

Patients with HF usually do not understand the cause and prognosis of their disease and rarely discuss the quality and end of life with the professionals involved in their care. Care for people with advanced progressive illnesses is currently prioritized by diagnosis rather than need. Patients with advanced HF should receive care that is proactive and designed to meet their specific needs.^18^

A chronic syndrome such as HF, which requires continuous treatment for an indeterminate period and is linked to aging and presence of comorbidities, is inexorably associated with worse quality of life.^13-15^

The present study had some limitations. This is a cross-sectional study where all evaluations were performed in a single day without follow-up of the population, leading to difficulty in establishing causal relationships between HF and loss of quality of life. Another limitation is related to the reduced number of HF cases assessed, which diminishes the power of the study, leading to the lack of statistical significance of some associations.

## Conclusions

Patients with HF have low quality of life independent of the syndrome phenotype. The quality of life evaluation in primary care could help identify patients who would benefit from a proactive healthcare program with more emphasis on multidisciplinary and social support. Therefore, strategies that can improve the quality of life of those patients and bring them greater benefits than the prolongation of life without associated quality are needed.

## References

[1] Morgan K, McGee H, Shelley E (2007). Quality of life assessment in heart failure interventions: a
10-year (1996-2005) review. Eur J Cardiovasc Prev Rehabil.

[2] Redfield MM, Jacobsen SJ, Burnett Jr JC, Mahoney DW, Bailey KR, Rodeheffer RJ (2003). Burden of systolic and diastolic ventricular dysfunction in the
community: appreciating the scope of the heart failure
epidemic. J Am Med Assoc.

[3] Vasan RS, Benjamin EJ, Levy D (1995). Prevalence, clinical features and prognosis of diastolic heart
failure: an epidemiologic perspective. J Am Coll Cardiol.

[4] Senni M, Tribouilloy CM, Rodeheffer RJ, Jacobsen SJ, Evans JM, Bailey KR (1991). Congestive heart failure in the community: a study of all
incident cases in Olmsted County, Minnesota. Circulation.

[5] Malki Q, Sharma ND, Afzal A, Ananthsubramaniam K, Abbas A, Jacobson G (2002). Clinical presentation, hospital length of stay, and readmission
rate in patients with heart failure with preserved and decreased left
ventricular systolic function. Clin Cardiol.

[6] Lewis EF, Johnson PA, Johnson W, Collins C, Griffin L, Stevenson LW (2001). Preferences for quality of life or survival expressed by patients
with heart failure. J Heart Lung Transplant.

[7] King D (1996). Diagnosis and management of heart failure in the
elderly. Postgrad Med J.

[8] McDonald K (2005). Current guidelines in the management of chronic heart failure:
practical issues in their application to the community
population. Eur J Heart Fail.

[9] Jorge AL, Rosa ML, Martins WA, Correia DM, Fernandes LC, Costa JA (2016). The prevalence of stages of heart failure in primary care: a
population-based study. J Card Fail.

[10] Jorge AJ, Rosa ML, Fernandes LC, Freire MD, Freire MD, Rodrigues RC (2011). Estudo da prevalência de insuficiência
cardíaca em indivíduos cadastrados no Programa Médico
de Família - Niterói. Estudo DIGITALIS: desenho e
método. Rev Bras Cardiol.

[11] McMurray JJ, Adamopoulos S, Anker SD, Auricchio A, Böhm M, Dickstein K, ESC Committee for Practice Guidelines (2012). ESC Guidelines for the diagnosis and treatment of acute and
chronic heart failure 2012: The Task Force for the Diagnosis and Treatment
of Acute and Chronic Heart Failure 2012 of the European Society of
Cardiology. Eur Heart J.

[12] Ciconelli RM, Ferraz MB, Santos W, Meinão I, Quaresma MR (1999). Tradução para a língua portuguesa e
validação do questionário genérico de
avaliação de qualidade de vida SF-36 (Brasil
SF-36). Rev Bras Reumatol.

[13] O'Mahony MS, Sim MF, Ho SF, Steward JA, Buchalter M, Burr M (2003). Diastolic heart failure in older people. Age Ageing.

[14] Riegel B, Carlson B, Glaser D, Romero T (2003). Changes over 6-months in health-related quality of life in a
matched sample of Hispanics and non-Hispanics with heart
failure. Qual Life Res.

[15] Jaarsma T, Halfens R, Abu-Saad HH, Dracup K, Stappers J, van Ree J (1999). Quality of life in older patients with systolic and diastolic
heart failure. Eur J Heart Fail.

[16] Lewis EF, Lamas GA, O'Meara E, Granger CB, Dunlap ME, McKelvie RS, CHARM Investigators (2007). Characterization of health-related quality of life in heart
failure patients with preserved versus low ejection fraction in
CHARM. Eur J Heart Fail.

[17] Masoudi FA, Rumsfeld JS, Havranek EP, House JA, Peterson ED, Krumholz HM, Cardiovascular Outcomes Research Consortium (2004). Age, functional capacity, and health-related quality of life in
patients with heart failure. J Card Fail.

[18] Murray SA, Boyd K, Kendall M, Worth A, Benton TF, Clausen H (2002). Dying of lung cancer or cardiac failure: prospective qualitative
interview study of patients and their carers in the
community. British Med J.

